# Supplementary material for: Freeze-and-Release Direct Optimization Method for Variational Calculations of Excited Electronic States
Source: J Chem Theory Comput. 2026 Mar 16;22(7):3571–84. doi: 10.1021/acs.jctc.5c01974 (PMC13085236; doi:10.1021/acs.jctc.5c01974)
Supplement: Supplementary file 1 [file ct5c01974_si_001.pdf]

# Supporting information for "Freeze-and-release direct optimization method for variational calculations of excited electronic states"

Yorick L. A. Schmerwitz,*,† Elli Selenius,‡ and Gianluca Levi*,¶,‡

†*Max-Planck-Institut für Kohlenforschung, 45470 Mülheim an der Ruhr, Germany*

‡*Science Institute of the University of Iceland, Reykjavík, Iceland*

¶*Department of Chemical and Pharmaceutical Sciences, University of Trieste, 34127 Trieste, Italy*

E-mail: schmerwitz@kofo.mpg.de; gianluca.levi@units.it

Table S1: Excitation energy (in eV) and charge transfer distance (in Å), $d^{CT}$, of charge transfer states of organic molecules obtained from orbital optimized calculations using FR-DO, DO-MOM, and SCF-MOM with PBE/aug-cc-pVDZ+sz, together with the theoretical best estimate (TBE) values of excitation energy.[1]

| Molecule | Sym. | TBE [a] | FR-DO | | DO-MOM | | SCF-MOM | |
|---|---|---|---|---|---|---|---|---|
| | | $\Delta$E | $\Delta$E | $d^{CT}$ | $\Delta$E | $d^{CT}$ | $\Delta$E | $d^{CT}$ |
| Aminobenzonitrile (ABN) | $A_1$ | 5.09 | 3.69 | 1.06 | 3.69 | 1.06 | 3.69 | 1.06 |
| Aniline | $A_1$ | 5.48 | 4.31 | 0.82 | 4.31 | 0.82 | 4.31 | 0.82 |
| Azulene | $A_1$ | 3.84 | 3.00 | 0.94 | 3.00 | 0.94 | 3.00 | 0.94 |
| | $B_2$ | 4.49 | 3.97 | 0.77 | 3.97 | 0.77 | 3.97 | 0.77 |
| Benzonitrile | $A_2$ | 7.05 | 6.57 | 1.04 | 5.96 | 0.58 | - | - |
| Benzothiadiazole (BTD) | $B_2$ | 4.28 | 3.08 | 1.19 | 3.08 | 1.19 | 3.08 | 1.19 |
| Dimethylaminobenzonitrile (DMABN) | $A_1$ | 4.86 | 3.53 | 1.55 | 3.53 | 1.56 | 3.54 | 1.56 |
| Twisted DMABN | $A_2$ | 4.12 | 3.56 | 2.04 | 3.56 | 2.04 | 3.56 | 2.05 |
| | $B_1$ | 4.75 | 4.21 | 1.75 | 4.21 | 1.75 | 4.21 | 1.75 |
| Dimethylaniline (DMAn) | $B_2$ | 4.40 | 3.82 | 1.08 | 3.82 | 1.08 | 3.82 | 1.08 |
| | $A_1$ | 5.40 | 4.17 | 1.33 | 4.17 | 1.33 | 4.18 | 1.33 |
| Hydrogen Chloride | $\Pi$ | 7.88 | 7.33 | 0.86 | 7.33 | 0.86 | 7.33 | 0.86 |
| p-Nitroaniline | $A_1$ | 4.39 | 3.26 | 2.05 | 3.26 | 2.05 | 3.26 | 2.05 |
| Nitrobenzene | $A_1$ | 5.39 | 4.13 | 1.46 | 4.13 | 1.46 | 4.13 | 1.47 |
| Nitrodimethylaniline (NDMA) | $A_1$ | 4.13 | 3.05 | 2.34 | 3.05 | 2.34 | 3.05 | 2.34 |
| Nitropyridine *N*-Oxide (NPNO) | $A_1$ | 4.10 | 2.73 | 1.72 | 2.73 | 1.72 | 2.73 | 1.72 |
| *N*-Phenylpyrrole (PP) | $B_2$ | 5.32 | 4.12 | 1.59 | 4.12 | 1.59 | - | - |
| | $A_1$ | 5.86 | 5.14 | 2.02 | 4.69 | 1.85 | 5.14 | 2.03 |
| Twisted PP | $B_2$ | 5.58 | 5.26 | 2.36 | 4.58 | 2.04 | 5.27 | 1.49 |
| | $A_1$ | 5.65 | 5.56 | 2.41 | 4.61 | 2.06 | 5.56 | 2.41 |
| | $A_2$ | 5.95 | 5.40 | 2.15 | 5.40 | 2.15 | 5.40 | 2.15 |
| | $B_1$ | 6.17 | 5.42 | 2.16 | 5.42 | 2.16 | 5.42 | 2.16 |
| Phthalazine | $A_2$ | 3.91 | 3.10 | 1.26 | 3.10 | 1.26 | 3.11 | 1.26 |
| | $B_1$ | 4.31 | 3.45 | 1.26 | 3.45 | 1.26 | 3.45 | 1.26 |
| Quinoxaline | $B_2$ | 4.63 | 3.48 | 1.25 | 3.48 | 1.25 | 3.48 | 1.25 |
| | $A_1$ | 5.65 | 4.56 | 0.62 | 4.56 | 0.62 | 4.56 | 0.62 |
| | $B_1$ | 6.22 | 5.15 | 1.24 | 5.15 | 1.24 | 5.15 | 1.24 |

[a] Theoretical best estimates obtained at the CCSDT/aug-cc-pVQZ level in ref. 1

Table S2: Computed saddle point order of charge transfer excited states of organic molecules at the initial guess, after constrained optimization, and at the target solution. The calculations use the PBE functional and the aug-cc-pVDZ+sz basis set. The values in parentheses are the number of negative eigenvalues with an absolute value bigger than 1 eV. Constrained optimization leads to a significant improvement in the estimated saddle point order for both a numeric eigendecomposition of the Hessian and a diagonal analytic approximation (preconditioner of eq 7 in the main text).

| Molecule | Sym. | Initial guess | | Constrained solution | | Final solution |
|---|---|---|---|---|---|---|
| | | Num. Hessian | Precond. (eq 7) | Num. Hessian | Precond. (eq 7) | Num. Hessian |
| Aminobenzonitrile (ABN) | $A_1$ | 2 (1) | 1 | 1 (1) | 1 | 1 |
| Aniline | $A_1$ | 4 (2) | 3 | 3 (1) | 3 | 3 |
| Azulene | $A_1$ | 2 (2) | 2 | 2 (2) | 2 | 2 |
| | $B_2$ | 3 (3) | 3 | 3 (3) | 3 | 3 |
| Benzonitrile | $A_2$ | 14 (13) | 3 | 6 (6) | 6 | 6 |
| Benzothiadiazole (BTD) | $B_2$ | 4 (3) | 1 | 1 (1) | 1 | 1 |
| Dimethylaminobenzonitrile (DMABN) | $A_1$ | 4 (1) | 1 | 1 (1) | 1 | 1 |
| Twisted DMABN | $A_2$ | 22 (18) | 1 | 6 (3) | 6 | 3 |
| | $B_1$ | 25 (22) | 2 | 5 (5) | 5 | 5 |
| Dimethylaniline (DMA) | $B_2$ | 11 (8) | 1 | 2 (1) | 2 | 2 |
| | $A_1$ | 8 (4) | 3 | 4 (2) | 3 | 3 |
| Hydrogen Chloride | $\Pi$ | 2 (1) | 1 | 2 (1) | 1 | 1 |
| p-Nitroaniline | $A_1$ | 31 (19) | 1 | 5 (4) | 5 | 3 |
| Nitrobenzene | $A_1$ | 18 (14) | 3 | 5 (4) | 5 | 4 |
| Nitrodimethylaniline (NDMA) | $A_1$ | 41 (26) | 1 | 6 (4) | 5 | 3 |
| Nitropyridine *N*-Oxide (NPNO) | $A_1$ | 13 (9) | 1 | 4 (3) | 4 | 2 |
| *N*-Phenylpyrrole (PP) | $B_2$ | 13 (8) | 1 | 3 (3) | 3 | 2 |
| | $A_1$ | 43 (36) | 2 | 9 (6) | 9 | 5 |
| Twisted PP | $B_2$ | 38 (31) | 1 | 10 (6) | 10 | 9 |
| | $A_1$ | 42 (35) | 2 | 12 (7) | 11 | 11 |
| | $A_2$ | 38 (29) | 2 | 11 (7) | 11 | 7 |
| | $B_1$ | 38 (34) | 3 | 12 (7) | 12 | 6 |
| Phthalazine | $A_2$ | 14 (12) | 1 | 2 (1) | 2 | 2 |
| | $B_1$ | 11 (8) | 2 | 3 (1) | 3 | 2 |
| Quinoxaline | $B_2$ | 6 (4) | 2 | 4 (2) | 4 | 2 |
| | $A_1$ | 5 (5) | 3 | 3 (3) | 3 | 3 |
| | $B_1$ | 10 (9) | 3 | 4 (4) | 4 | 4 |
| Avg. abs. deviation | | 13.6 (9.7) | 1.7 | 1.2 (0.7) | 1.1 | |
| Max. abs. deviation | | 38 (31) | 9 | 6 (4) | 6 | |

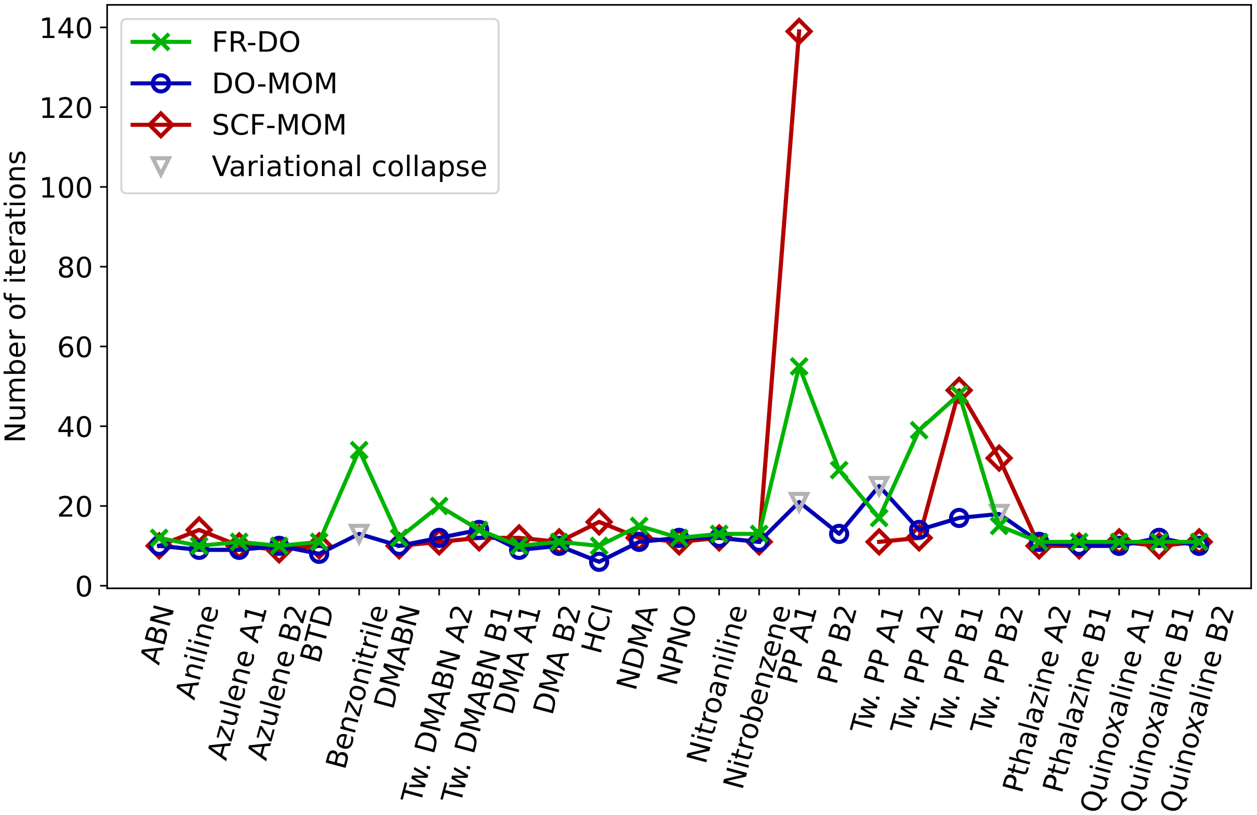


Figure S1: Number of iterations taken by FR-DO (green crosses), DO-MOM (blue circles), and SCF-MOM (red diamonds) to converge each excited state in the set of intramolecular charge transfer states. Variational collapses are indicated by gray triangles.

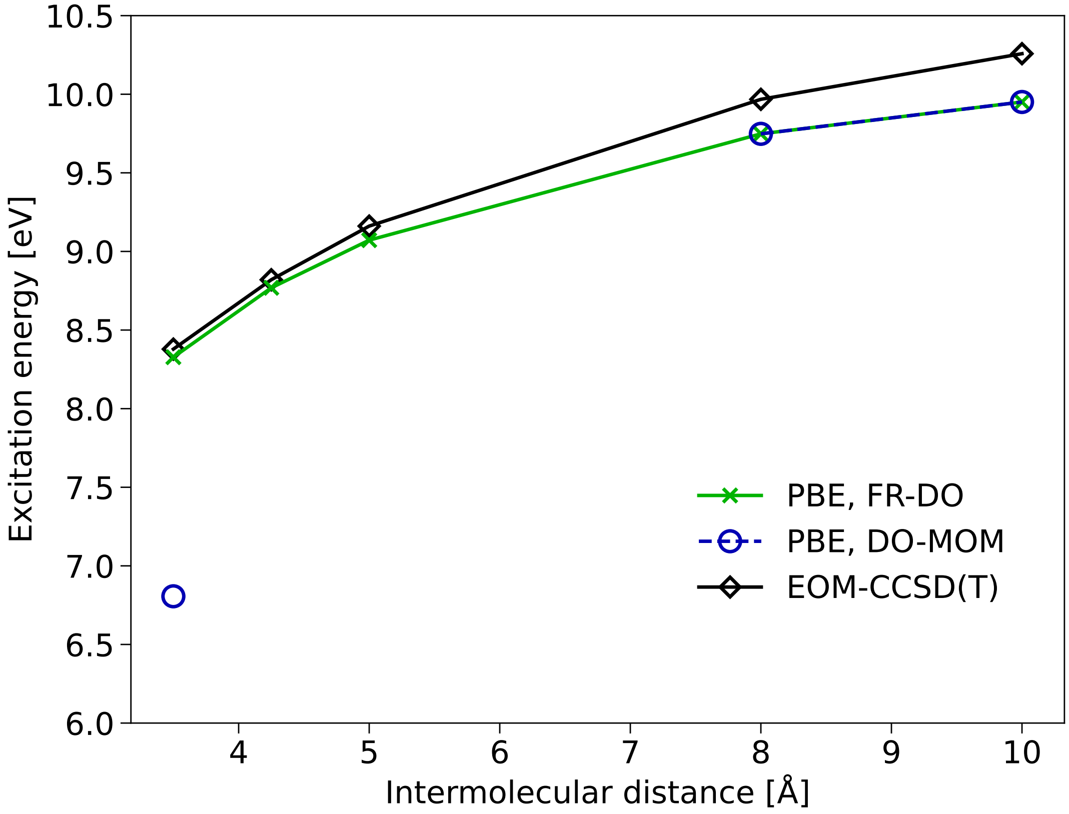


Figure S2: Excitation energy of the ammonia-fluorine dimer as a function of the distance between the molecular fragments obtained in FR-DO (green crosses) and DO-MOM (blue circles) calculations with PBE/cc-pVDZ+sz, and taken from published EOM-CCSD(T) calculations[2] (black squares). The FR-DO energy curve agrees well with the many-body results. DO-MOM shows a variational collapse at an intermolecular distance of 3.5 Å and does not converge within 333 iterations until a distance of 8 Å is reached, above which it converges to the same solution as FR-DO.

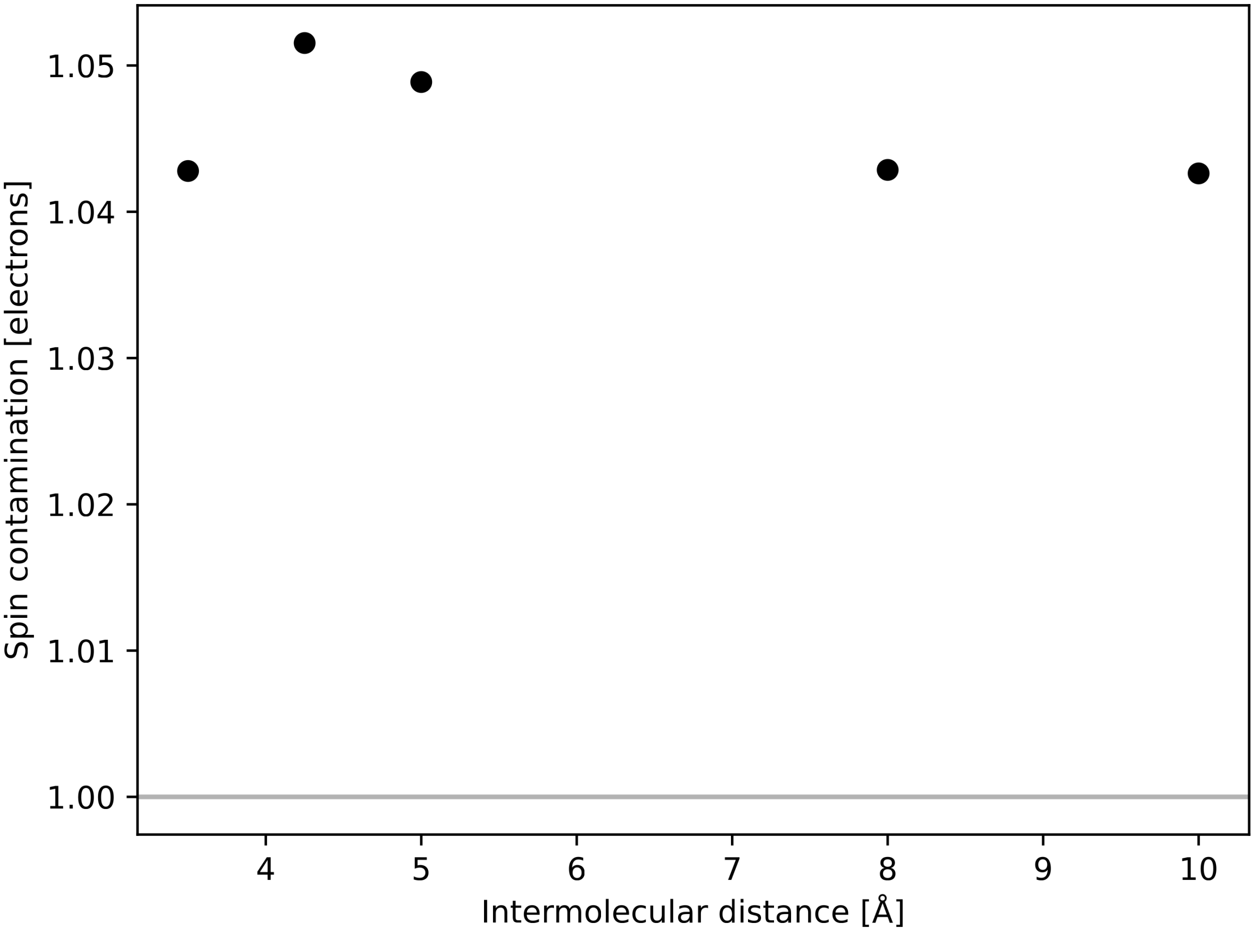


Figure S3: Spin contamination of an intermolecular charge transfer excited state of the ammonia-fluorine dimer with respect to intermolecular distance obtained in FR-DO calculations using PBE/cc-pVDZ+sz. The spin density is computed as the integral of the negative part of the spin density, $\rho_s(\mathbf{r}) = \rho_\uparrow(\mathbf{r}) - \rho_\downarrow(\mathbf{r})$ (see eq 13 in the main text). A spin contamination value of 1 is highlighted by the gray horizontal line, indicating the value expected for a spin-mixed excited state solution.

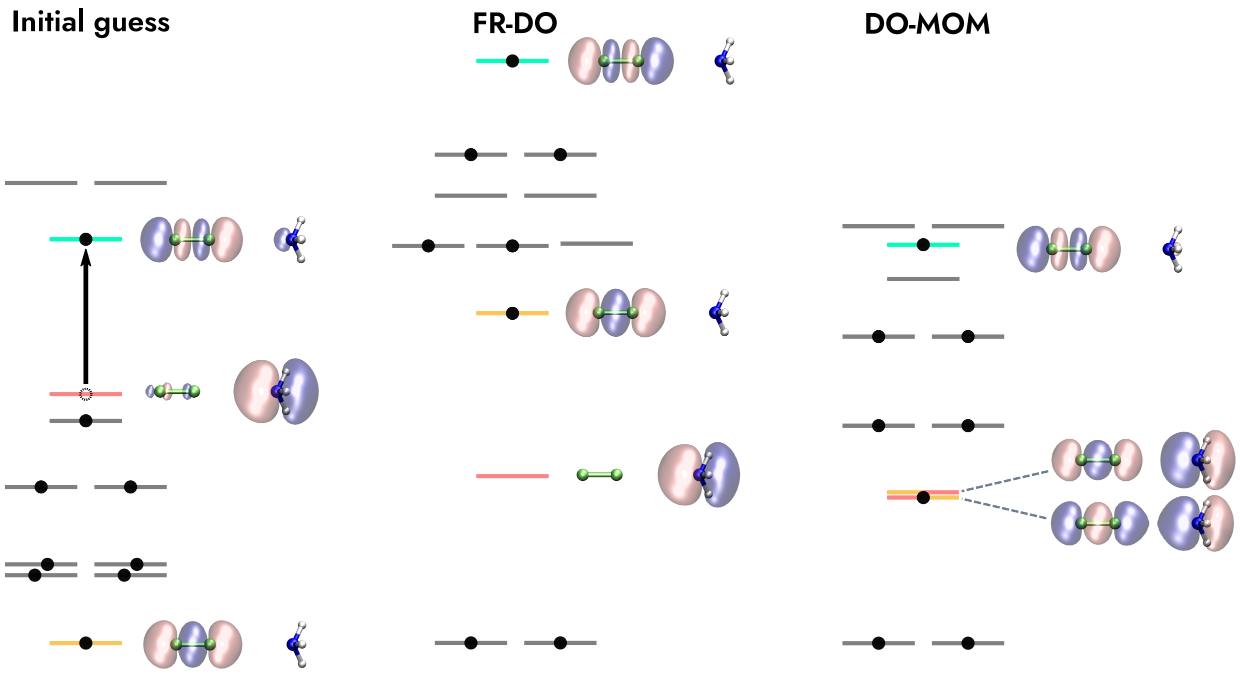


Figure S4: Molecular orbitals of the initial guess, the FR-DO solution, and the DO-MOM solution for the ammonia-fluorine dimer at an intermolecular distance of 3.5 Å. The orbitals are visualized with isosurface values of $\pm 0.08\,\text{Å}^{-3}$.

# References


(1) Loos, P.-F.; Comin, M.; Blase, X.; Jacquemin, D. Reference energies for intramolecular charge-transfer excitations. *Journal of Chemical Theory and Computation* **2021**, *17*, 3666–3686.

(2) Bogo, N.; Stein, C. J. Benchmarking DFT-based excited-state methods for intermolecular charge-transfer excitations. *Phys. Chem. Chem. Phys.* **2024**, *26*, 21575–21588.
